# The role of MR imaging in detection of hepatic iron overload in patients with cirrhosis of different origins

**DOI:** 10.1186/1471-230X-10-13

**Published:** 2010-01-27

**Authors:** Edyta Szurowska, Katarzyna Sikorska, E Iżycka-Świeszewska, Tomasz Nowicki, Tomasz Romanowski, Krzysztof P Bielawski, Michał Studniarek

**Affiliations:** 1 Department of Radiology, Medical University of Gdansk, Debinki 7, 80-211 Gdansk, Poland; 2 Department of Infectious Diseases, Medical University of Gdansk, Debinki 7, 80-211 Gdansk, Poland; 3 Department of Pathology, Medical University of Gdansk, Debinki 7, 80-211 Gdansk, Poland; 4 Molecular Diagnostics Division, Department of Biotechnology, Intercollegiate Faculty of Biotechnology, University of Gdansk and Medical University of Gdansk, Kladki 24, 80-822 Gdansk, Poland

## Abstract

**Background:**

There are many pathological conditions with hepatic iron overload. Classical definite diagnostic methods of these disorders are invasive and based on a direct tissue biopsy material. For the last years the role of MR imaging in liver diagnostics has been increasing. MRI shows changes of liver intensity in patients with hepatic iron overload. Changes in MR signal are an indirect consequence of change of relaxation times T2 and T2*, that can be directly measured.

The purpose of the study was to evaluate usefulness of MR imaging in the detection of hepatic iron overload in patients with cirrhosis of different origins.

**Methods:**

MR imaging at 1.5T was prospectively performed in 44 patients with liver cirrhosis who had undergone liver biopsy with histopathological assessment of hepatic iron deposits. In all patients the following sequences were used: SE, Express, GRE in T2 and T1-weighted images. Signal intensity (SI) was measured on images obtained with each T2 weighted sequence by means of regions of interest, placed in the liver and paraspinal muscles. The correlation between iron overload, histopathological score, serum ferritin and SI ratio was analyzed.

**Results:**

In 20 patients with iron overload confirmed by the biopsy, the liver parenchyma demonstrated lower signal intensity than that of paraspinal muscles. This effect was visible only in 8 patients with hepatic iron overload in Express T2-weighted images. Higher signal intensity of liver than that of skeletal muscles on GRE - T2 weighted images was noted in 24 patients with cirrhosis and without elevated hepatic iron concentration. We observed a correlation between low and high iron concentration and liver to muscle SI ratio.

**Conclusion:**

MR imaging is a useful and fast noninvasive diagnostic tool for the detection of liver iron overload in patients with cirrhosis of different origins.

Liver to muscle SI ratio in GRE-T2-weighted sequence facilitates to differentiate patients with low and high degree of hepatic iron overload, which correlates with the origin of liver cirrhosis.

## Background

There are many entities in human pathology with hepatic iron overload. There is controversy regarding nomenclature of these states, however, the classic example of primary iron overload is defined as hereditary hemochromatosis (HH). HH is one of the most common genetic disorders and the second most frequent metabolic liver disease - with the frequency of 3-8 cases/1000 people [1-3]. *HFE *gene is involved in most cases of hereditary hemochromatosis type 1 due to its two recessive missense mutations Cys282Tyr and His63Asp [4]. The inborn defect of iron metabolism leads to progressing iron deposition in the liver, pancreas, heart and endocrine glands. There are also other types of primary hemochromatosis associated with different genetic anomalies, including juvenile HH, *TFR2*-associated, ferroportin-associated and neonatal HH [5].

Secondary/acquired iron overload diseases develop as a consequence of iron loading anemia, thalassemia and sideroblastic anemia. Interestingly, iron overload, usually of a low grade is common in many chronic liver diseases. The examples are: chronic viral hepatitis especially type C, alcoholic liver disease, non-alcoholic fatty liver disease (NAFLD), hepatic porphyria and cirrhosis. In inflammatory disorders, the accumulation of iron may correlate with the level of inflammation and stage of disease. Moreover, there is a possibility of a coincidence of HH with above mentioned liver diseases and in some cases only genetic examination yields the final diagnosis.

The biochemical markers of the iron metabolism disorders include elevated concentration of iron and ferritin and transferrin saturation in plasma. However, these parameters are not always specific for body iron load [6]. A confirmation of overimposed tissue iron deposition is the gold standard in HH diagnosis. For years, quantitative spectrophotometric analysis of the biopsy sample has been the best method for its direct assessment in the liver [7]. Another widely accepted method is based on histological indirect assessment of the hepatic iron stores on tissue slides stained with Prussian blue- Pearl's stain, identifying ferric iron. There are many grading systems for hepatic iron stores. Most commonly used in routine histopathology practice is Scheuer's system and Deugnier's system for the experimental studies [8]. However, the efficacy of this exam is limited in the patients with liver cirrhosis due to relatively low sample representativity. The iron concentration in such cases can be uneven because of irregular fibrosis and nodular remodeling of the organ [9].

Optimization of the standards and introduction of non-invasive assessment of body iron accumulation by means of magnetic resonance imaging (MRI) has been recently pursued. Modification of the signal intensity in magnetic resonance imaging in presence of iron, especially in T2-weighted images is the key factor in clinical application of this diagnostic method. Changes in MR signal are an indirect consequence of change of relaxation times T2 and T2*, that can be measured directly.

MRI allows both the recognition of excessive iron accumulation in liver tissue and the evaluation of the level of iron overload [10,11] In the recent studies a good correlation between MR technique and biopsy results with LIC measurement was observed [11-13].

In everyday practice two basic methods of hepatic iron content have been used: older and better known signal/ratio technique is based on signal intensity of liver and paraspinal muscle in 5 GRE sequences and on correlation between these values and calibration curve of iron concentration in a biopsy sample. However, this technique today is almost completely overcome by quantitative techniques, consisting of the direct calculation of T2 or T2* by fitting an appropriate decay model to the average signal intensity at various echo times [12-15].

Direct conversion of T2 or T2* in quantitative iron content is possible by establishing of calibration curves for quantitative iron overload assessment for these multiecho SE or GRE approaches.

MR technique is a modality of choice especially in patients presenting contradictions to liver biopsy or in cases when the quantitative assessment of liver iron concentration cannot be performed. This method can also have an important clinical meaning in patients with irregular iron deposition in liver cirrhosis. The technique may enable assessment of differences in iron deposition in patients with cirrhosis of different origin. In the recent studies a correlation between iron overload and pathogenic HCV and alcohol overdosing has been proved [16-18].

According to our knowledge, the presented study is the first research to use MR imaging in iron deposition assessment in cirrhotic patients of different origin.

The aim of the study was to assess the efficacy of MRI examination in the detection of liver iron overload in patients with liver cirrhosis of different origins. Liver signal intensity and liver-to-muscle SI ratio was estimated and compared with histologically assessed level of liver iron concentration [19,20] and serum ferritin concentration.

## Methods

The study was performed on 44 consecutive patients with liver cirrhosis. They were chosen from the total number of 300 patients with hepatic pathology examined with MRI in our department (period 2004-2008) who underwent the liver biopsy with histopathological iron load assessment in the liver specimen and no adjustments to chelation treatment were made between examinations. The period between two investigations was maximum 31 days.

The examined group consisted of 27 men and 17 women aged from 22 to 81 years (median 64 years).

Iron deposition in the oligobiopsy specimen was evaluated according to Scheuer's grading scale on tissue sections stained with Pearl's method [19].

The MRI was performed in 11 patients due to hemochromatosis suspicion whereas in 33 people hepatocellular carcinoma in course of cirrhosis was suspected.

In 11 above mentioned patients hemochromatosis was suggested because of abnormal biochemical tests of iron turnover (iron concentration > 28 micromol/l with increased transferring saturation > 50% or increased ferritin concentration > 400 ng/ml) and histopathologically proved iron deposition within the hepatic tissue. In all these patients *HFE *gene molecular analyses were conducted.

Genetic testing for C282Y, H63D and S65C *HFE *gene mutations was performed by PCR and restriction fragment length polymorphism (PCR-RFLP) analysis after extraction of genomic DNA from blood leukocytes, collected from 11 patients. The method was previously described [21]. Homozygotic C282Y and combined heterozygotic C282Y/H63D *HFE *mutations were considered as a genetic manifestation of HH.

MRI examination was performed with 1.5 T MR scanner (Eclipse- Picker). The sequences SE, Express, GRE in T1 and T2-weighted image were used in three planes. All these sequences were carried out on breath-hold images with layer width of 5-6 mm, gap: 1 mm, matrix 226 × 256, FOV: 34 - 40 cm, with the body coil type. The precise description of T2-weighted sequences used to iron overload assessment are presented in table 1.

**Table 1:** Magnetic resonance T2-weighted sequences used in the study.

Type of sequence	TR (ms)	TE (ms)	PA (°)
GRE- T2 weighted	50	15	30

Express-T2-weighted	18000	80	90

TR - repetition time, TE - echo time, PA - pulse angle.

In cancer suspected cases the additional multiphase exam after i.v. contrast medium administration was done.

The MR image analysis of iron overload within the liver was semi-quantitative. The measurements of liver signal intensity (liver SI) were made in 5 random points of area 2-4 cm^2^(100-200 pixels) far from big blood vessels and within the back muscles (muscle SI) in three random points. Signal intensity (SI) was measured on images obtained with each T2-weighted sequence by the means of regions of interest, placed in the liver and paraspinal muscle to obtain the liver-to-muscle SI ratio. Next, the comparative evaluation of liver and muscle SI was made and the liver-to-muscle SI ratio was counted.

**Figure 1:**
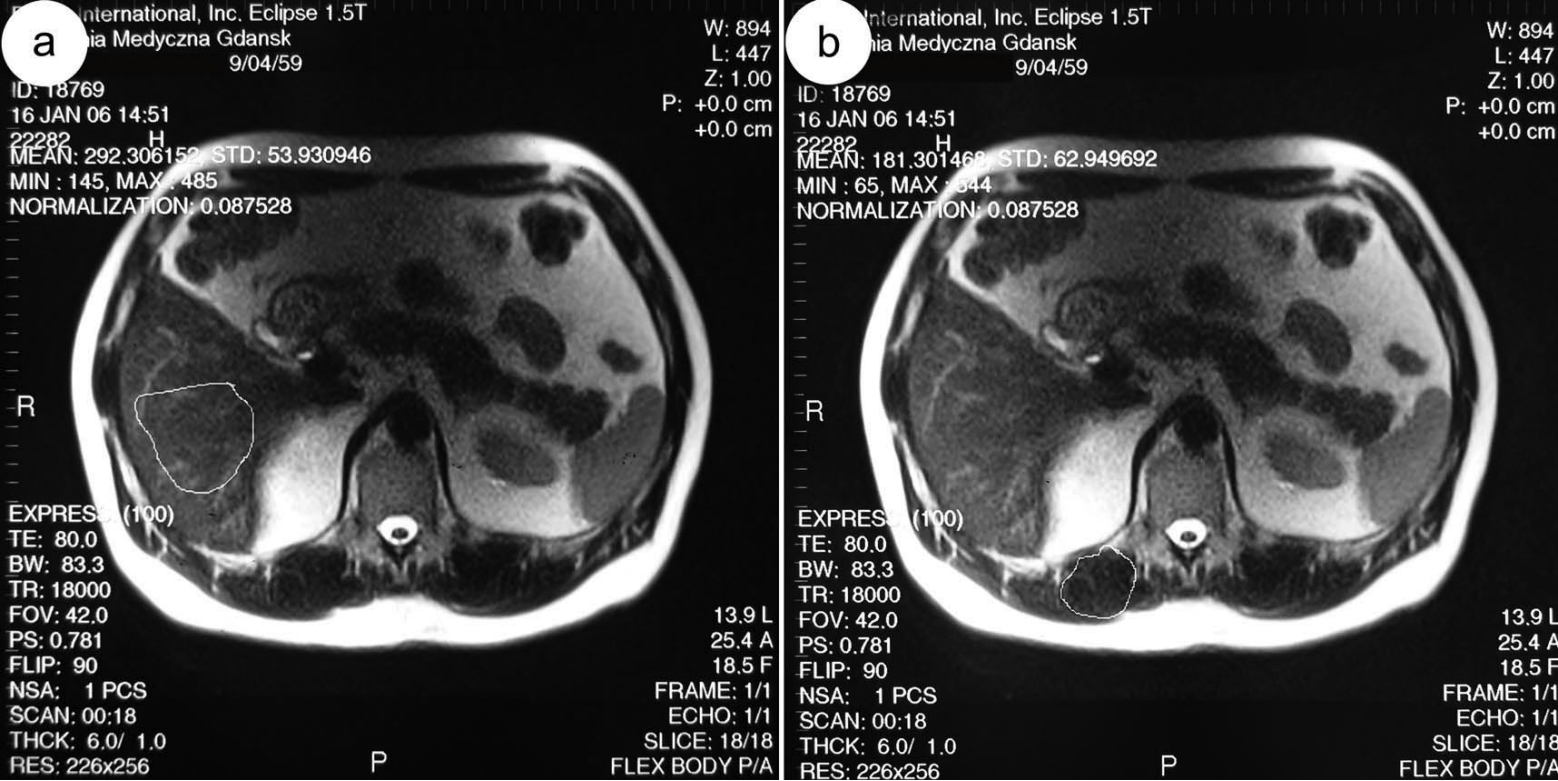
**47-year-old man with normal hepatic iron level confirmed by the biopsy**. Axial MR T2-weighted images performed on gradient echo sequence show normal liver. The signal intensity of liver (fig.1a - the signal intensity of liver is 292) is higher than that of paraspinal muscles (fig.1b - the signal intensity of paraspinal muscles is 181).

**Figure 2:**
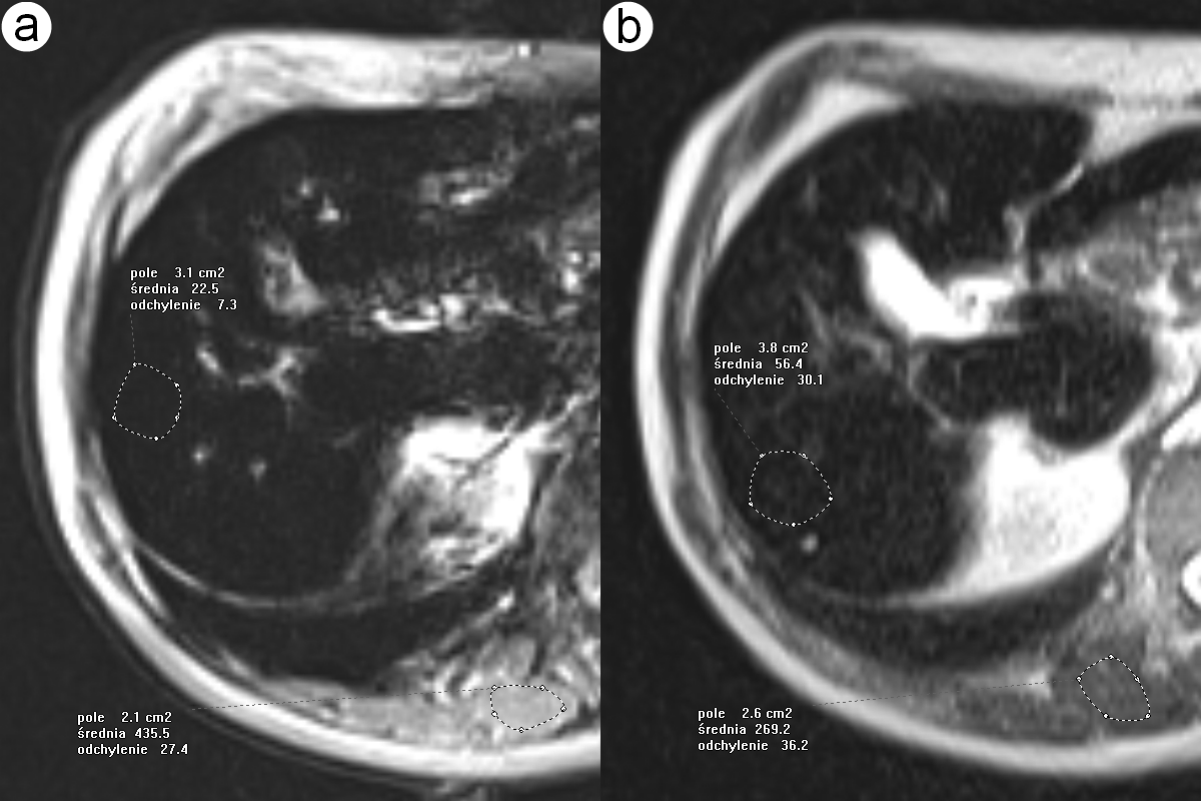
**68-year-old man with cirrhosis and hemosiderosis confirmed by the biopsy**. Axial MR T2-weighted images performed on gradient echo (fig.2a) and Express (fig.2b) sequences show typical findings for hepatic iron overload. The signal intensity of liver (accordingly 23 and 56) is lower than that of paraspinal muscles (accordingly 436 and 269).

**Figure 3:**
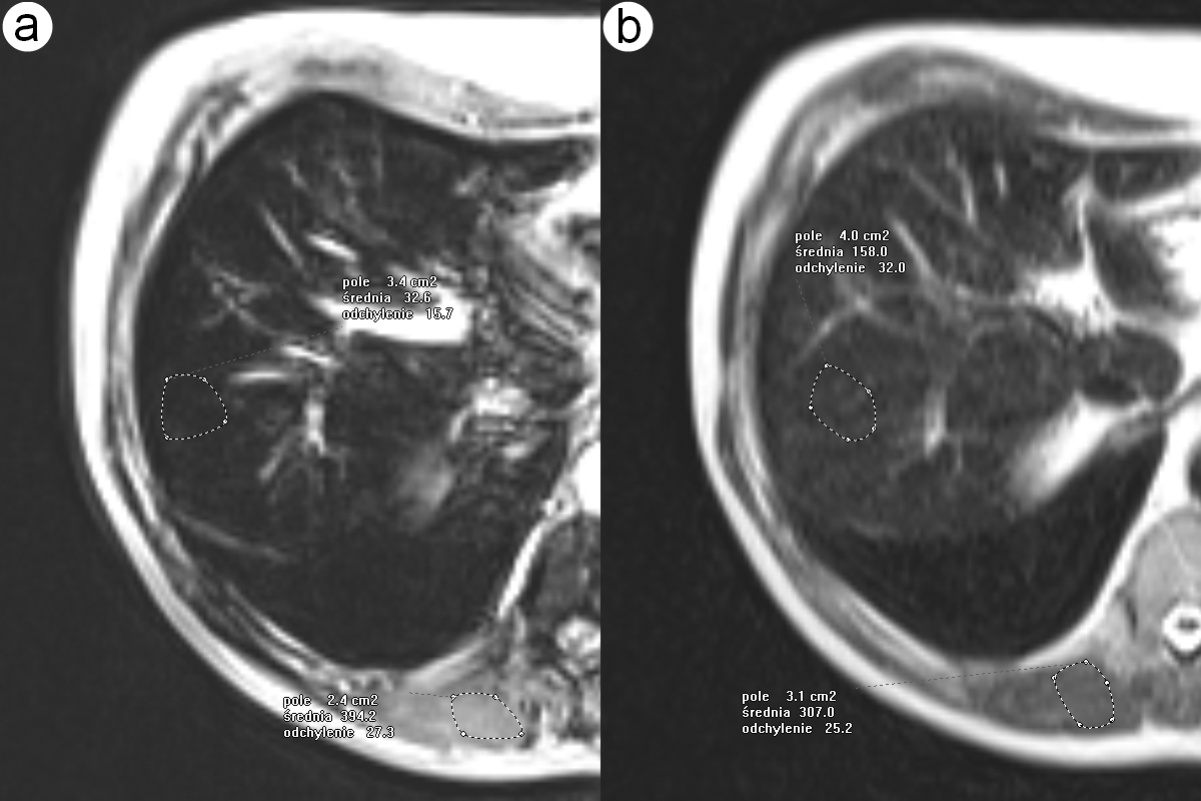
**45-year-old man with cirrhosis and hemochromatosis confirmed by the biopsy and genetic examination**. Axial MR T2-weighted images performed on gradient echo (fig.3a) and Express (fig.3b) sequence show typical findings for hepatic iron overload. The signal intensity of liver (accordingly 33 and 158) is higher than that of paraspinal muscles (accordingly 394 and 307).

The next analyses concerned the examination of a relation between liver SI and the level of liver iron deposits assessed by histopathological methods. The liver-to muscle SI ratio was also compared with the histopathological grade of iron load [10,20,22,23]. According to the literature, the marker of HH was liver SI lower than SI of back muscles (fig. 1, 2, 3).

The difference between liver SI and liver-to muscle SI ratio in cirrhotic patients of various origin (HH, HCV-related cirrhosis, HCV - HBV related cirrhosis and HBV related cirrhosis) has been evaluated. Additionally the correlation between ferritin level and Scheuer's grading scale was calculated.

To prove that sequences proposed by our team, Express and GRE, are reproductive and that signal intensity used in these methods depends on iron concentration, we preformed additional examination of 23 phantoms made of fragmented pork liver. 20 of the phantoms contained increasing iron (III) chloride concentrations and 3 were used as validation. After subjecting fragment of liver used to drying at 104°C for 24 hours, the phantoms' wet-weight (ww) iron concentration were converted to dry-weight (dw) concentrations.

The research was approved by Independent Bioethic Committee for Scientific Research of Medical University of Gdansk (NKEBN-443-2004) and patients gave their written informed consent to participate.

For statistic analysis program STATISTICA 8 (StatSoft Inc, Tulsa, OK, USA), Mann-Whitney *U *test and Spearman's rank correlation coefficient were used. ROC curve was calculated with MedCalc 9.6.2.0 (MedCalc Software bvba, Belgium).

## Results and Discussion

All patients were classified as a group A of cirrhosis in Child-Pugh score. In liver biopsy of 20 patients, the increased iron load was found with a grade 1-4 in Scheuer's scale. These patients were described as a subgroup A made of 15 men and 5 women at the age 22-73 (median 55 years). More than a half of the subgroup A - 11 people included the patients with clinical suspicion of HH and with high levels of iron and ferritin (168-11100 ng/ml) or high value of transferrin saturation in serum (40-90%, mean 77,17%). In this group 5 patients appeared to be carriers of the *HFE *gene mutations typical for HH type 1 (3 homozygotes C282Y/C282Y, 2 combined heterozygotes C282Y/H63D). In 4 out of 11 patients other *HFE *mutations were found - 2 homozygotes H63D/H63D and 2 heterozygotes H63D/WT), which also predispose for iron accumulation [24,25]. In two patients postinflammatory HCV cirrhosis was recognized.

In the patients with genetically confirmed HH (5 out of 9) and in patients with *HFE *gene mutation H63D/H63D (2 out of 9 persons), the intense iron accumulation within the liver was observed with 3 or 4 grade in Scheuer's scale (7/9). In patients with heterozygotic for H63D *HFE *gene mutation (2 cases) and in only cirrhotic patients the low and intermediate liver iron overload was found (1-2 grade in Scheuer's scale). The data are presented in table 2. In the second subgroup - B, there were 24 patients (12 men and 12 women) at the age 32-81 years (median 69) with liver cirrhosis without signs of liver iron overload (grade 0).

**Table 2:** Group A description - 20 patients with iron metabolism disturbances.

Iron content: points by Scheuer's grading scale	diagnosis	number of patients
4	Hereditary hemochromatosis type 1 - *HFE *gene mutation	3

3	Hereditary hemochromatosis type 1 - *HFE *gene mutation	2
	
	*HFE *gene mutation (2 homozygotes H63D/H63D)	2
	
	HCV-related cirrhosis C	2

2	*HFE *gene mutation (2 heterozygotes H63D/WT)	2
	
	HCV-related cirrhosis (2) and HCV/HBV related cirrhosis (4)	6

1	HCV/HBV related cirrhosis	3

**Figure 4:**
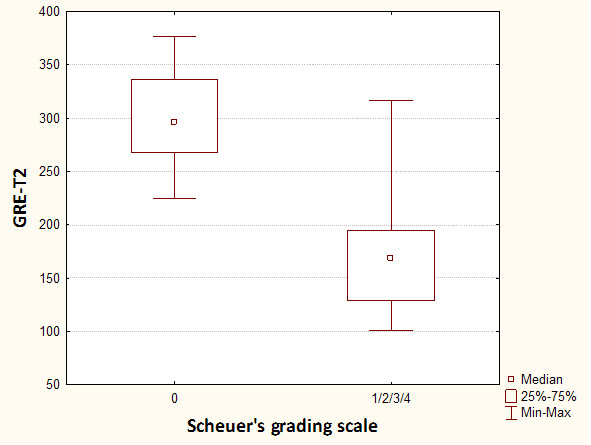
**Box-and-whisker plots show mean liver signal intensity in patients without (0 pts in Scheuer's grading scale) and with (1-4 pts in Scheuer's grading scale) hepatic iron overload using gradient echo MR sequence**.

**Figure 5:**
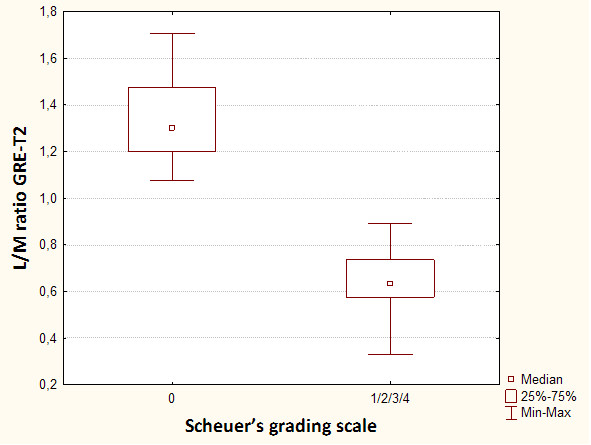
**Box-and-whisker plots show mean liver to muscle signal intensity ratio in patients without (0 pts in Scheuer's grading scale) and with (1-4 pts in Scheuer's grading scale) hepatic iron overload using gradient echo MR sequence**.

**Figure 6:**
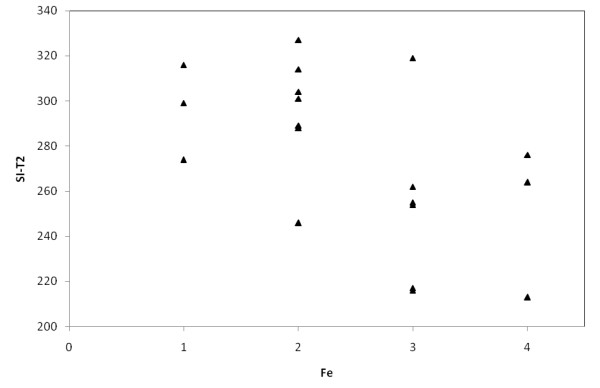
**Relationship between liver signal intensity in Express sequence in T2 weighted images (SI-T2) and iron content in liver by semiquantitative histological Scheuer's grading scale (Fe)**.

**Figure 7:**
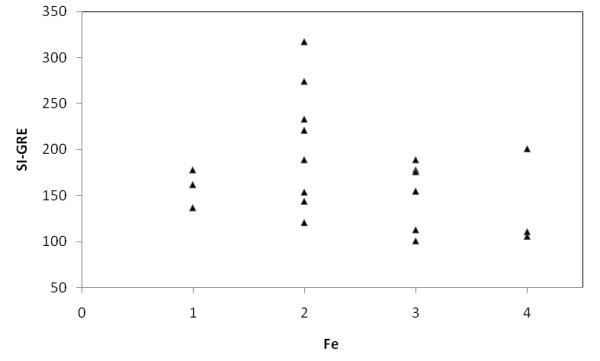
**Relationship between liver signal intensity in gradient echo sequence in T2 weighted images (SI-GRE) and iron content in liver by semiquantitative histological Scheuer's grading scale (Fe)**.

**Figure 8:**
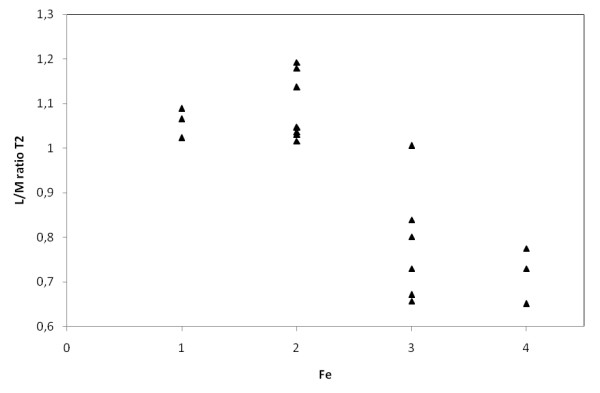
**Relationship between the liver-to-muscle SI ratio in Express sequence in T2 weighted images (L/M ratio T2) and iron content in liver by semiquantitative histological Scheuer's grading scale (Fe)**.

**Figure 9:**
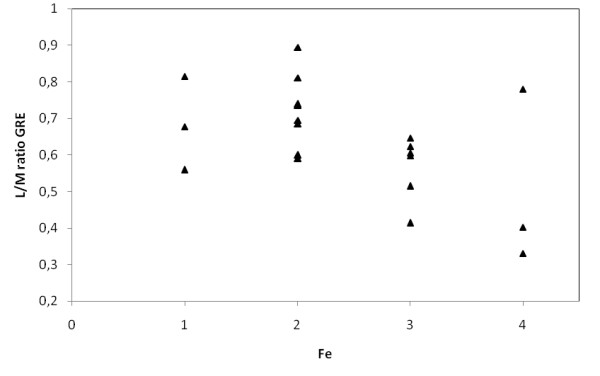
**Relationship between the liver-to-muscle SI ratio in gradient echo sequence in T2 weighted images (L/M ratio GRE) and iron content in liver by semiquantitative histological Scheuer's grading scale (Fe)**.

**Figure 10:**
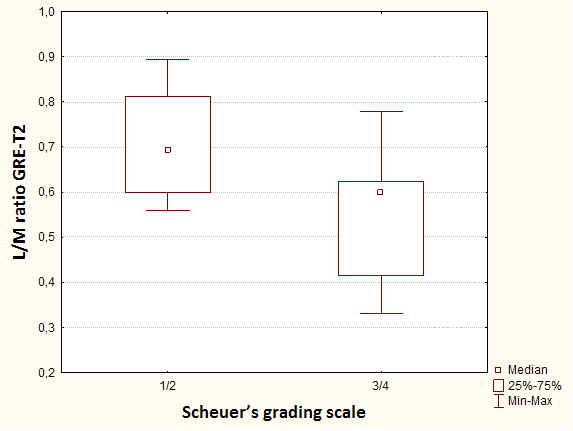
**Box-and-whisker plots show mean liver to muscle signal intensity ratio in two groups of patients - with low to medium (1-2 pts in Scheuer's grading scale) and high (3-4 pts in Scheuer's grading scale) hepatic iron overload using gradient echo MR sequence**.

**Figure 11:**
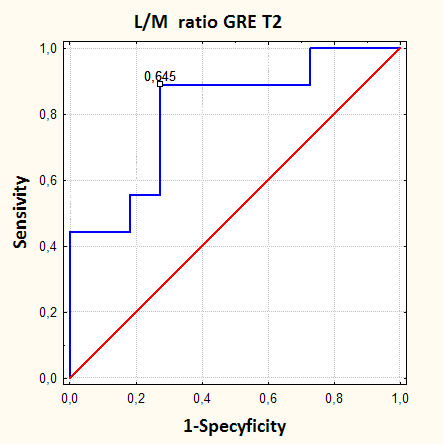
**Graphs showing receiver-operating characteristics (ROC) curve analysis to determine a threshold of liver to muscle SI ratio suitable for differentiating patients with low to medium (1-2 pts in Scheuer's grading scale) and high (3-4 pts in Scheuer's grading scale) hepatic iron overload using gradient echo MR sequence**. The area under the curve corresponds to accuracy of this method. The sensitivity of this method for the threshold of liver to muscle SI ratio - 0.645 is 89%.

In MRI in T2-weighted image lower liver SI than back muscles was observed in Express sequence in 8 cases with iron overload, whereas in GRE sequence in all patients with iron overload - all cases subgroup A. Average liver SI in GRE (fig. 4) in patients with iron overload was lower than in the group of patients with cirrhosis without overload - subgroup B (173 vs 298). The difference was statistically significant. The analogous correlation but not significant was noted in Express sequence (277 vs 294). The relative liver to muscle SI ratio was significantly different in group with iron overload (liver to muscle SI ratio < 0.896) and in patients without iron turnover disturbances (liver to muscle SI ratio > 1.08). The results are shown in fig. 5. The parameter liver-to-muscle SI ratio has high values as marker of diagnostic efficacy and it is almost equal to certainty (all diagnostic efficacy markers: sensivity, specificy and efficacy, PPV and NPV are 100%). Moreover we noticed faint relation between liver SI in Express sequence and liver iron concentration, namely: higher concentration - lower SI (fig. 6). This tendency is stronger in examination with GRE sequences (fig. 7) than in Express (fig. 6). The evident correlation between liver-to-muscle SI ratio and histological scale was observed - the higher iron concentration, the lower liver-to-muscle SI ratio. This relation is shown worse in Express than in GRE (fig. 8, 9). The biggest difference is visible between low and intermediate iron concentration (1,2 grade) and higher overload (3,4 grade) which is shown in fig. 10. Based on the values of liver-to-muscle SI the cut-off value of the index was found on ROC curve, which the most effectively divides patients into low and high liver iron overload (fig 11). For the threshold at 0,645 method sensitivity was 89%, specificity 73%, PPV-73% and NPV- 89%. The field under the curve shows efficacy of 80%.

**Figure 12:**
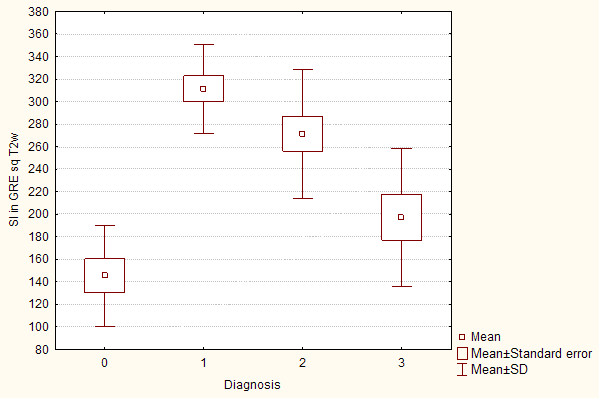
**Box-and-whisker plots show mean liver SI in gradient echo sequence in T2 weighted images of patients with cirrhosis of different origins (0-HH, 1- HBV related cirrhosis, 2- HCV-HBV related cirrhosis and 3- HCV related cirrhosis)**.

**Figure 13:**
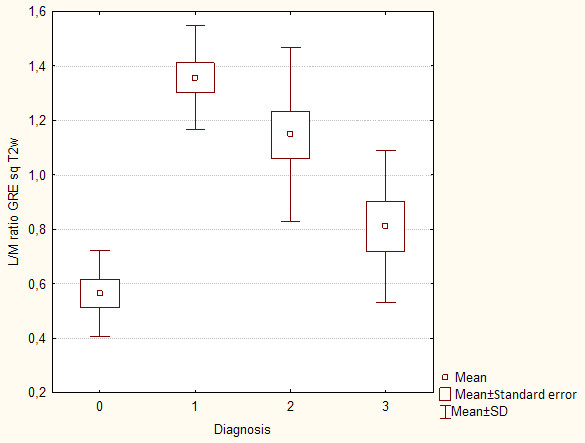
**Box-and-whisker plots show mean liver-to-muscle ratio in gradient echo sequence in T2 weighted images of patients with cirrhosis of different origins (0-HH, 1- HBV related cirrhosis, 2- HCV-HBV related cirrhosis and 3- HCV related cirrhosis)**.

Differences in liver SI (fig. 12) and liver-to-muscle SI ratio (fig. 13) in GRE sequence were found depending on pathogenic factor for cirrhosis. Differences between HH, HCV/HBV related cirrhosis and HBV related cirrhosis groups are statistically significant (*p *< 0,01).

**Figure 14:**
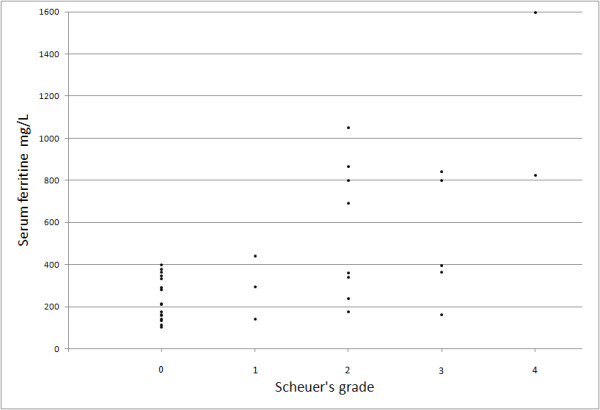
**Correlation between serum ferritin and Scheuer's grading scale**. Point for ferritin level of 11000 μg/L and grade 4 outplaced in figure.

There was a statistic correlation between serum ferritin level and Scheuer's grading scale (fig. 14 r_s _= 0,62, p < 0,001).

**Figure 15:**
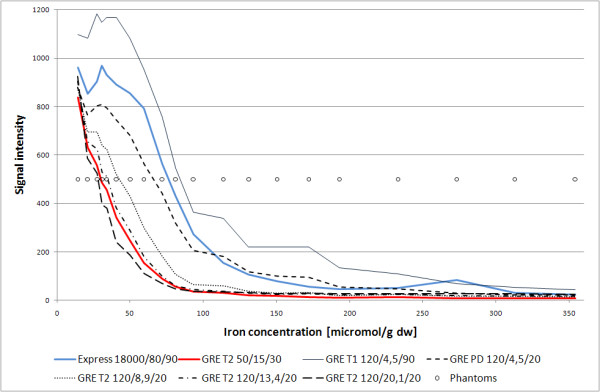
**Relationship between SI and iron concentration in phantoms in different sequences**.

In experimental part of our study (fig. 15) we used phantoms, which were a cheap, easy and acceptable method of MR imaging standardization. We obtained a good correlation between iron concentration in pork liver and SI gained from sequences proposed in this study and by Gandon [26] which could be used as a reference method [27]. This correlation allowed to acknowledge our methodology of examination as reliable and reproducible. The GRE sequence was suitable for iron concentrations from 10 up to 150 micromol/g dw and Express sequence from 50 to 300 micromol/g dw. By using both sequences detection of iron concentrasion from 10 up to 300 micromol/g dw was possible.

In general, in the course of chronic diseases iron deposition within the liver leads to the increase of the existing oxidative stress, intensificates fibrogenesis, the development of cirrhosis and increased risk of HCC. Iron overload is found on histopathological exam in 32-78% of the patients with long standing hepatic cirrhosis [9,28]. In the presented study iron deposition was observed in 45% of the patients with cirrhosis. In this group only 1/3 of the cases had genetically proved hemochromatosis. Ludwig et al. diagnosed hepatic iron overload in 32% of the cases in their study conducted on 447 patients with liver cirrhosis, but inherited HH only in five [8]. Above differences are caused by the type of analyzed patients populations, a selective choice of patients with HH and an advanced stage of cirrhosis in our series where the patients were suspected of HCC. MRI is an accepted method used for iron detection in liver and other organs [11,22,29].

In MR imaging the increased liver iron concentration prolongs T2 relaxation time and lowers liver signal intensity, resulting in a typical hypointensive, dark liver. The opinions about the influence of fibrosis and fatty change grade on iron concentration in histopathological and MRI liver images are divided, however, in majority of studies no correlations were found [20]. The correlation between MR image and biochemical markers of liver iron storing is usually good [11,23].

Blood ferritin concentrations is widely used as a marker of liver iron overload, but it has poor specificity in patients with inflammation (for example hepatitis C) [27,30,31]. In our study we stated correlation between ferritin level and iron assessed by biopsy. Very high values of ferritin correlate with liver iron deposits mainly in liver cirrhosis that is a consequence of hereditary hemochromatosis (HH). In cirrhosis of different origin iron accumulation is not so intense, often accompanies only severe necroinflammation and ferritin values are lower compared to cases with HH. Events that occur in end-stage liver disease may cause decrease of ferritin values.

Alustiza et al. used not definite measurements of liver SI for quantitative assessment of iron deposition in the liver and a relative value expressed by liver to paraspinal muscle SI ratio, reaching high detection efficacy of MRI method [23]. Kaltwasser reported very high correlation (r = 98%) between T2 liver signal intensity and iron concentration in 10 patients confirmed with a biopsy [29]. The group of Bonkovsky et al. found the best correlation between LIC and the natural logarithm ratio of the liver signal intensity and the background noise [32]. Gandon et al. reported that the effective assessment of iron concentration within the liver parenchyma (LIC) is possible in the range of 80-300 mmol/g. For values of more than 300, the quantitative evaluation was impossible due to the complete loss of signal [11].

In our study, including previous analyses on phantoms, we did not observe the complete loss of signal, even in the patients with very high iron concentration (4 grade in Scheuer's scale) [33]. Multitude of used comparative methods in MRI to detect iron overload in liver is an effort of methodological standarization of this method, but shows its relatively low reproducibility.

In recent years a significant development of quantitative techniques for assessment of liver iron deposition has been observed which use direct calculation of T2 and T2*based on multiecho SE or GRE sequence and comparing calibration curves. Andersen et al. stated negative log-linear correlation between Liver T2* and HIC of 0,93 in liver without fibrosis [14]. The relationship between R2 and iron concentration was nearly linear. St Pierre et al. in their research on large population (over 100 patients) showed a curvilinear R2 relationship between R2 and HIC, marked using biopsy, in clinically significant range of iron deposition in the liver [12]. In another study with 102 patients with iron overload and 13 healthy people, Wood and colleagues compared the relationship between iron deposits and R2 and R2* [13]. HIC was assessed by biopsy in 22 patients with liver iron overload and both R2 and R2* values correlated closely with HIC, R2 had curvilinear realationship. Positano et al. proposed a global method to increase the accuracy of T2* assessment using software phantom resembling real image data, which allows to reduce the operator dependence and sampling errors [15]. This method can be successfully used to assess borderline liver iron overload and monitoring therapy.

In presented study we used a well-known scheme - the liver-to-muscle SI ratio and we received a correlation between the-liver-to-muscle SI ratio and a degree of iron overload (fig. 8, 9). Alustiza et al. did not achieve the compliance between LIC assessed with spectrophotometry and MRI only in 13% of patients, our results are similar (11% of patients) [22,32]. Some authors advises to use gradient echo sequence, which is concordant with our analyses [11,23,32]. The presented study shows that GRE sequence is more sensitive (100% of sensitivity) than Express one (40% of sensitivity).

In our study the correlation between iron concentration assessed seminquantitatively and signal intensity is visible but weakly expressed (fig. 6, 7). It is caused probably by few reasons: a big difference in a patient's individual magnetic susceptibility, the lack of technical calibration of equipment and different superficial coil used in MRI exam. To avoid incorrect measurements, coming from irregularity of the magnetic field on the border of phase array coil, we used a body coil.

The significant difference is noted between liver-to-muscle SI ratio between low grade iron overload (1,2 Scheuer's grade) and high overload (3,4 Scheuer's grade). Relative liver signal intensity - liver-to-muscle SI ratio lower than 0.645 is a marker characteristic for the advanced iron overload with sensitivity of 89% and accuracy of 80% (fig. 11). This value divides our patients into two clinical groups: with hepatic iron deposits according to 1,2 grade in Scheuer's scale and HH (hepatic iron deposits contributing to 3,4 grade in Scheuer's scale). On these grounds we can say that it is possible to diversify the causes of pathological liver iron overload.

A difference between liver SI or liver-to-muscle SI ratio and cause of liver cirrhosis has been proved: the highest iron accumulation (the lowest signal of the liver) was observed in group of patients with hereditary hemochromatosis, lower in patients with HCV/HBV - related cirrhosis and HCV-related cirrhosis and lack of pathological accumulation (the highest signal of the liver) in patients with HBV-related cirrhosis (fig. 12, 14). A major tissue iron accumulation is observed in hereditary hemochromatosis type 1 homozygotic C282Y and compound heterozygotic C282Y/H63D HFE gene mutations leading to dysfunction of the HFE protein. In other chronic viral or toxic liver diseases, iron overload may be less pronounced as a result of synergy between both genetic predisposition and influence of other pathogenic factors [34]. In alcoholic and viral liver injury iron overload accompany severe necroinflammatory process [30]. Alcohol as a promoter of iron accumulation may act through increase of iron absorption or influence on hepcidin and transferrin expression [35-37]. Patients with chronic hepatitis C present more severe hepatic iron accumulation compared to chronic hepatitis B [16,17]. Iron overload is described in 17-30% cases of patients with chronic hepatitis C and HCV-related cirrhosis [30]. In our study 4 from 14 patients with HCV-related cirrhosis (29% of cases) and 7 from 9 patients with coinfection HCV/HBV-related cirrhosis (77% of cases) had recognized acquired iron-overload disorder. Probably impairment of hepcidin expression, that is a main iron metabolism regulatory protein is caused just by HCV infection [38]. Presumbly in cases with hepatitis B (HBV) and C (HCV) viruses iron loading if present is a result of pathogenic influence of HCV. Coinfection with HCV/HBV viruses usually is associated with higher risk of unfavourable course, treatment difficulties and rapid progression of liver fibrosis. Evaluation of both reasons and intensity of iron overload as a potential prognostic factor in that coinfection needs detailed analysis.

There are some limitations of our study. First of all we did not compare liver SI and the liver-to-muscle SI ratio to quantitative, real HIC, but to semiquantitative histological evaluation. Secondly, in time of new generation equipment with possibility of performing multiecho SE and GRE sequences with direct conversion of T2 and T2* in quantitative iron content, our methodology is not modern, but still signal/ratio technique is widely used.

However information achieved thanks to it can lead to new direction in research of liver cirrhosis of different origins and allows to assess iron accumulation using non-invasive technique.

## Conclusions

In summary, MRI with use of gradient echo sequence in T2-weighted image is very useful and non-invasive method for detection of liver iron overload in patients with cirrhosis of different origins. GRE-T2-weighted sequence is characterized by a higher sensitivity than Express in evaluation of iron deposition in the liver. MRI enables to diversify between low and advanced liver iron overload which correlates with the origin of liver cirrhosis.

## Abbreviations

dw: dry-weight; GRE: gradient echo; HH: hereditary hemochromatosis; HIC: hepatic iron concentration; LIC: liver iron concentration; MR: magnetic resonance; MRI: magnetic resonance imaging; NPV: negative predictive value; PCR: polymerase chain reaction; PPV: positive predictive value; ROC: receiver operating characteristic; SE: spin echo; SI: signal intensity; ww: wet-weight.

## Competing interests

The authors declare that they have no competing interests.

## Authors' contributions

ES has substantial contributions to study design, data collection and interpretation, statistic analysis and manuscript preparation. KS has contributed to study design, data collection and interpretation, manuscript preparation and founds collection. EIS has contributed to data collection and interpretation and manuscript preparation. TN has contributed to data collection and interpretation, statistic analysis and manuscript preparation. TR and KPB have performed molecular genetic studies. MS has contributed to study design and has coordinated research team. All authors have read and approved the final manuscript.

## Pre-publication history

The pre-publication history for this paper can be accessed here:

http://www.biomedcentral.com/1471-230X/10/13/prepub
